# Serum level of soluble CX3CL1/fractalkine is elevated in patients with polymyositis and dermatomyositis, which is correlated with disease activity

**DOI:** 10.1186/ar3761

**Published:** 2012-03-06

**Authors:** Fumihito Suzuki, Tetsuo Kubota, Yasunari Miyazaki, Kinya Ishikawa, Masashi Ebisawa, Shunsei Hirohata, Takashi Ogura, Hidehiro Mizusawa, Toshio Imai, Nobuyuki Miyasaka, Toshihiro Nanki

**Affiliations:** 1 Department of Medicine and Rheumatology, Graduate School of Medical and Dental Sciences, Tokyo Medical and Dental University, 1-5-45 Yushima, Bunkyo-ku, Tokyo 113-8519, Japan; 2 Department of Integrated Pulmonology, Graduate School of Medical and Dental Sciences, Tokyo Medical and Dental University, 1-5-45 Yushima, Bunkyo-ku, Tokyo 113-8519, Japan; 3 Department of Neurology and Neurological Science, Graduate School of Medical and Dental Sciences, Tokyo Medical and Dental University, 1-5-45 Yushima, Bunkyo-ku, Tokyo 113-8519, Japan; 4 Department of Rheumatology and Infectious Diseases, Kitasato University School of Medicine, 1-15-1 Kitasato, Minami-ku, Sagamihara, Kanagawa 252-0374, Japan; 5 Department of Respiratory Medicine, Kanagawa Cardiovascular and Respiratory Center, 6-16-6 Tomioka-higashi, Kanazawa-ku, Yokohama, Kanagawa 236-0051, Japan; 6 KAN Research Institute, 3F, Kobe MI R&D Center, 6-7-3 Minatojima-minamimachi, Chuo-ku, Kobe, Hyogo 650-0047, Japan

## Abstract

**Introduction:**

Polymyositis (PM) and dermatomyositis (DM) are chronic inflammatory muscle diseases, in which chemokines are thought to contribute to inflammatory cell migration into muscle. In this study, we retrospectively analyzed the expressions of CX3CL1/fractalkine and its corresponding receptor, CX3CR1, in muscle and lung with interstitial lung disease (ILD) of PM patients and DM patients, and determined the correlation between serum soluble CX3CL1 level and disease activity.

**Methods:**

Expressions of CX3CL1 and CX3CR1 in muscle and lung tissue were analyzed by immunohistochemistry. Serum CX3CL1 concentrations were measured by ELISA. For evaluation of patients' disease activity, serum creatinine kinase, manual muscle testing, and the alveolar-arterial oxygen pressure difference were used independently.

**Results:**

CX3CL1 was expressed on infiltrated mononuclear cells and endothelial cells in muscle affected by PM and DM and in lung with ILD, whereas CX3CR1 was expressed on some CD4^+ ^T cells, a majority of CD8^+ ^T cells, and most macrophages in muscle, and on infiltrated mononuclear cells in the lung. Serum soluble CX3CL1 was significantly higher in PM patients and DM patients than in healthy controls. The CX3CL1 level was correlated with serum creatinine kinase and manual muscle testing score. In patients with PM and DM with ILD, serum CX3CL1 was also correlated with alveolar-arterial oxygen pressure difference. Furthermore, CX3CL1 was significantly decreased after conventional treatment.

**Conclusions:**

The interaction between CX3CL1 and CX3CR1 might contribute to the inflammatory cell infiltration into affected muscle and lung with ILD in PM patients and DM patients. Serum CX3CL1 level could be a surrogate marker of disease activity.

## Introduction

Polymyositis (PM) and dermatomyositis (DM) are chronic inflammatory diseases affecting skeletal muscle with infiltration of mononuclear cells, such as CD4 and CD8 T cells and macrophages [1]. The infiltrating cells might contribute to the pathogenesis of PM and DM by releasing cytokines and cytotoxic molecules, such as TNF, perforin, and granzyme [2,3].

Patients with PM and patients with DM commonly show proximal muscle weakness, and some patients exhibit complication of interstitial lung disease (ILD), which may be associated with rapidly progressive respiratory insufficiency leading to death [4,5]. Serum creatinine kinase (CK) is helpful in evaluating both myositis activity and its response to the treatment. In some patients, however, serum levels of CK do not correspond to clinical disease activity [6,7]; moreover, the CK level does not correlate with ILD activity. Determining a new biomarker that is correlated with the activity of myositis and ILD is therefore useful.

Chemokines are known as important factors for cellular recruitment into inflamed tissues [8]. CX3CL1/fractalkine, a unique CX3C chemokine, exists in two forms: a membrane-bound form and a soluble form [9-11]. CX3CR1, a unique receptor for CX3CL1, is expressed on peripheral blood CD4^+ ^and CD8^+ ^T cells that produce cytotoxic molecules and type 1 cytokines, as well as on monocytes [12-14]. Since cytotoxic T cells and macrophages invade the affected muscle in patients with PM and in patients with DM [15], interaction between CX3CL1 and CX3CR1 may contribute to the inflammatory cell migration into muscle.

We previously reported that CX3CL1 was expressed in affected muscle in a murine model of experimental autoimmune myositis (EAM) and that CX3CR1 was expressed on the infiltrated CD4^+ ^and CD8^+ ^T cells and macrophages in the muscle [16]. We also found that the treatment of EAM mice with anti-CX3CL1 mAb significantly improved the myositis [16]. The interaction between CX3CL1 and CX3CR1 has therefore been suggested to perhaps probably play an important role in the pathogenesis of murine EAM. In this study, we aimed to determine the expressions of CX3CL1 and CX3CR1 in muscle and lung with ILD in PM patients and DM patients and to investigate the correlation between serum soluble CX3CL1 level and disease activity.

## Materials and methods

### Patients and samples

Serum, muscle, or lung specimens were collected from a total of 38 patients with PM and with DM who were admitted to Tokyo Medical and Dental University Hospital or to collaborating medical centers between 2001 and 2009 because of an active newly diagnosed form or flaring up of the disease. Tables 1 and 2 show the characteristics of the patients in this study. PM and DM were diagnosed according to the criteria developed by Bohan and Peter [17,18].

**Table 1:** Characteristics of the patients with polymyositis/dermatomyositis for serum analysis

	Healthy control serum (*n *= 20)	PM/DM patient serum (*n *= 29)
Number of PM/DM		7/22
Number of males/females	9/11	9/20
Age (years)	38.6 ± 2.1	55.3 ± 2.5
Duration of the disease (months)		21.6 ± 8.2
Number of cases of new onset/flare		23/6
Number of antinuclear antibody-positive		9 (*n *= 22)
Number of anti-Jo-1 antibody-positive		8 (*n *= 22)
Serum creatinine kinase (IU/l)^a^		4,063.8 ± 1,466.9
Manual muscle testing score		39.0 ± 1.4 (*n *= 18)
Number of patients with ILD		19
AaDO_2 _with ILD		31.5 ± 7.9 (*n *= 19)
Treatment at time of blood sampling		
Untreated		19
Prednisolone alone		4
Prednisolone and cyclosporine		5
Prednisolone and methotrexate		1

Data presented as *n *or mean ± standard error of the mean. Antinuclear antibody was analyzed by immunofluorescence assay. The titer of anti-Jo-1 antibody was investigated by enzyme immunoassay. Although in some patients anti-dsDNA (*n *= 17), anti-RNP (*n *= 13), anti-Scl-70 (*n *= 4), and anti-SS-A (*n *= 12) antibodies and rheumatoid factor (*n *= 16) were analyzed, they were all negative. AaDO_2_, alveolar-arterial oxygen pressure difference; ILD, interstitial lung disease; PM/DM, polymyositis/dermatomyositis. ^a^Normal range: male < 197 IU/l, female < 180 IU/l.

**Table 2:** Characteristics of the patients with polymyositis/dermatomyositis for muscle and lung analysis

	PM/DM patients (*n *= 11)	
	
	Muscle (*n *= 5)	Lung (*n *= 6)
Number of PM/DM	2/3	3/3
Number of males/females	2/3	1/5
Age (years)	60.4 ± 5.3	59.8 ± 4.3
Duration of the disease (month)	17.6 ± 13.4	20.8 ± 13.2
Number of cases of new onset/flare	4/1	3/3
Number of antinuclear antibody-positive	1 (*n *= 4)	1 (*n *= 5)
Number of anti-Jo-1 antibody-positive	0 (*n *= 4)	1 (*n *= 5)
Serum creatinine kinase (IU/l)^a^	2,946.4 ± 1,195.4	141.4 ± 57.4 (*n *= 5)
Manual muscle testing score	54.3 ± 5.8 (*n *= 4)	NA
Number of patients with ILD	4	6
AaDO_2 _with ILD	NA	22.7 ± 6.6 (*n *= 5)
Treatment at time of biopsy		
Untreated	4	4
Prednisolone alone	0	2
Prednisolone and cyclosporine	1	0

Data presented as *n *or mean ± standard error of the mean. Antinuclear antibody was analyzed by immunofluorescence assay. The titer of anti-Jo-1 antibody was investigated by enzyme immunoassay. Although in some patients, anti-dsDNA (*n *= 1 and *n *= 2 for muscle and lung samples, respectively), anti-RNP (*n *= 1 and *n *= 2), anti-Scl-70 (*n *= 1 and *n *= 2), and anti-SS-A (*n *= 1 and *n *= 4) antibodies and rheumatoid factor (*n *= 2 and *n *= 4) were analyzed, they were all negative. AaDO_2_, alveolar-arterial oxygen pressure difference; ILD, interstitial lung disease; NA, not available; PM/DM, polymyositis/dermatomyositis. ^a^Normal range: male < 197 IU/l, female < 180 IU/l.

ILD was diagnosed by clinical findings as follows: fine crackles, exertional dyspnea, nonproductive cough, and reticular shadow on chest radiographs or ground-glass opacity on chest high-resolution computed tomography. The disease activity was defined as symmetrical and proximal muscle weakness with serum CK elevation, or gradual or rapidly progressive ILD accompanied by the findings described above. Patients who also exhibited other connective tissue diseases, malignancies, infections, and hemophagocytic syndrome were excluded from this study. The experimental protocol was approved in advance by the Ethics Committee of Tokyo Medical and Dental University.

### Immunohistochemistry

Muscle specimens from five patients (two PM patients and three DM patients) were obtained by muscle biopsy from deltoid or biceps brachii. One of the five patients was also analyzed for the serum sample. Three control muscle samples were obtained from patients with increased serum CK of unknown etiology. No abnormal histological changes, such as small fiber or inflammatory cell infiltration, were observed in the control specimens. The specimens were frozen immediately in chilled isopentane precooled in liquid nitrogen, and then cryostat sections 6 μm thick were prepared. To analyze CX3CL1 expression, after fixing in cold acetone, the sections were treated with 1.5% H_2_O_2 _in methanol for 15 minutes, and then with 5% normal goat serum in PBS for 30 minutes. The sections were incubated with 5 μg/ml mouse anti-human CX3CL1 mAb (51637.11; R&D Systems, Minneapolis, MN, USA) or isotype-matched control mAb at 4°C overnight. CX3CL1 expression was detected using the Envision+ kit (Dako, Carpinteria, CA, USA). Diaminobenzidine chromogen and a buffered substrate were used for visualization. The sections were counterstained with hematoxylin. For double-staining with CD4, CD8, or CD68, as well as CX3CR1, the sections were incubated with 10 μg/ml mouse anti-human CD4 mAb (34930; R&D Systems), 1:20 diluted mouse anti-human CD8 mAb (HIT8a; BD Biosciences, San Jose, CA, USA), 1:100 diluted mouse anti-human CD68 mAb (KP-1; Dako), or control mAbs at 4°C overnight. Subsequently, the samples were incubated with 5 μg/ml Alexa Fluor^® ^488-conjugated goat anti-mouse IgG (Molecular Probes, Eugene, OR, USA) for 1 hour at room temperature. For CX3CR1 staining, the sections were incubated with 5 μg/ml rabbit anti-human CX3CR1 antibody (Santa Cruz Biotechnology, Santa Cruz, CA, USA) or normal rabbit IgG for 2 hours at room temperature. Next, the samples were incubated with 5 μg/ml Alexa Fluor^® ^568-conjugated goat anti-rabbit IgG (Molecular Probes) for 1 hour at room temperature. The slides were examined using fluorescent microscopy (BZ-Analyzer; Keyence, Tokyo, Japan).

Lung specimens from six patients (three PM patients and three DM patients) were obtained by video-assisted thoracic surgery. One of the six patients was also analyzed for the serum sample. Two control lung tissues were obtained by lobectomy for removal of primary lung tumors. No histological evidence of interstitial disease was found in any of the resected tissue samples. Paraffin-embedded tissues were deparaffinized and treated with 0.3% H_2_O_2_, followed by incubation with Protein Block (Dako) and an avidin/biotin blocking kit (Vector, Burlingame, CA, USA). The sections were incubated with 2 μg/ml mouse anti-CX3CL1 mAb (81513; R&D Systems) or 2 μg/ml rabbit anti-CX3CR1 antibody (Abcam, Cambridge, MA, USA) at 4°C overnight. After washing, they were incubated with biotin-conjugated rabbit anti-mouse IgG (Dako) or goat anti-rabbit IgG (Dako) for 30 minutes at room temperature, followed by the addition of peroxidase-conjugated streptavidin (Nichirei, Tokyo, Japan) for 5 minutes. Peroxidase activity was visualized using diaminobenzidine. Sections were counterstained with hematoxylin.

### Measurement of serum level of CX3CL1

Serum samples were collected from a total of 29 patients (seven PM patients and 22 DM patients) and 20 healthy controls. Fourteen of the patients (three PM patients and 11 DM patients) were traceable after the treatment (six patients with prednisolone alone, two patients with prednisolone + intravenous cyclophosphamide, two patients with prednisolone + cyclosporin, three patients with prednisolone + tacrolimus, and one patient with prednisolone + intravenous immunoglobulin). The median time of observation was 44.5 months. The concentration of serum CX3CL1 was measured using an ELISA kit (DuoSet; R&D Systems) in accordance with the manufacturer's protocol. All samples were stored at -80°C before use. The samples were assayed in duplicate.

### Assessment of muscle strength

To evaluate the muscle weakness of patients with myopathy, manual muscle testing (MMT) was measured using a 0-point to 5-point scale. The technique is summarized elsewhere [19]. We selected 10 muscles including deltoid, biceps, triceps, quadriceps, and hamstring, both right and left sides, and the maximum point score was 50, which indicates full muscle strength. The MMT data and serum sample were available from 18 patients (six PM patients and 12 DM patients).

### Statistical analysis

Differences in the levels of CX3CL1 between patients with PM, patients with DM, and healthy controls were examined for statistical significance using the Kruskal-Wallis test. To compare serum CX3CL1 before and after treatment, the Wilcoxon signed-rank test was employed. Pearson correlation coefficient was used to examine the relationship between the level of CX3CL1 and serum CK, MMT score, or the alveolar-arterial oxygen pressure difference (AaDO_2_). All data are expressed as mean ± standard error of the mean. The difference between groups was considered significant when *P *< 0.05.

## Results

### Expressions of CX3CL1 and CX3CR1 in muscle in PM and DM patients

**Figure 1:**
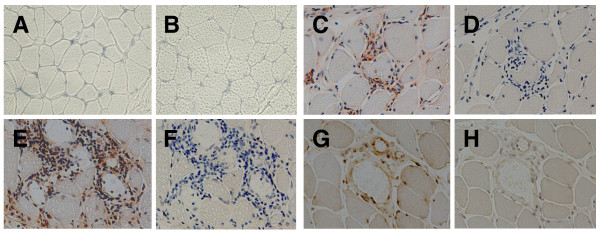
**CX3CL1 expressions in affected muscle of patients with polymyositis and patients with dermatomyositis**. Muscle specimens from **(A, B) **controls, **(C, D, G, H) **polymyositis (PM) patients, and **(E, F) **dermatomyositis (DM) patients were stained with anti-CX3CL1 mAb (A, C, E, G) or an isotype-matched control mAb (B, D, F, H). Original magnification, ×200.

We examined the expression of CX3CL1 in the affected muscle tissues in patients with PM and patients with DM by immunohistochemistry. CX3CL1 was not detected in control samples (Figure 1A). In contrast, CX3CL1 was expressed on infiltrated mononuclear cells in the muscle of those with PM and with DM (Figure 1C and 1E, respectively). CX3CL1 was also expressed on endothelial cells in the inflamed tissues of those with PM and with DM (Figure 1G and data not shown, respectively).

**Figure 2:**
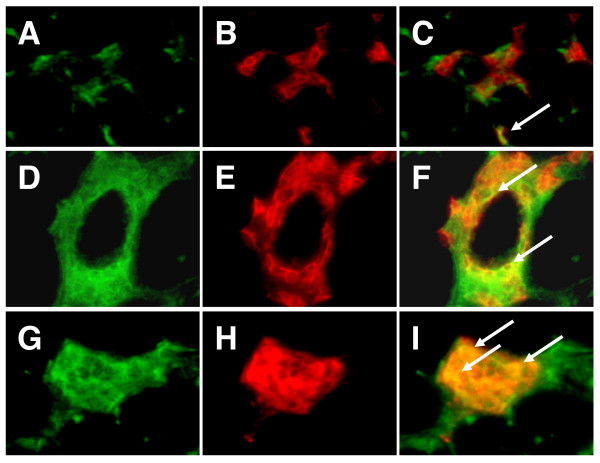
**CX3CR1 expressions in affected muscle of patients with polymyositis**. The muscle tissues of polymyositis (PM) patients were double-stained with CD4, CD8, or CD68, as well as CX3CR1, and were analyzed with fluorescent microscopy: **(A) **CX3CR1, **(B) **CD4, **(C) **merged (A) and (B), **(D) **CX3CR1, **(E) **CD8, **(F) **merged (D) and (E), **(G) **CX3CR1, **(H) **CD68, and **(I) **merged (G) and (H). Arrows indicate double-positive cells. Original magnification, ×400.

We next examined CX3CR1 expression on the infiltrated mononuclear cells in patients with PM and patients with DM by double immunohistochemical staining. In PM, a few CD4^+ ^T cells expressed CX3CR1 (Figure 2A to 2C). The majority of CD8^+ ^T cells and most CD68-positive macrophages expressed CX3CR1 in the inflamed tissues of patients with PM (Figure 2D to 2F and 2G to 2I, respectively). Similar CX3CR1 expression on the infiltrated mononuclear cells was observed in the muscle of DM patients (data not shown). Table 3 shows the expression of CX3CL1 and CX3CR1 in the muscle tissue from each of the patients.

**Table 3:** Expression of CX3CL1 and CX3CR1 in the muscle tissue from each of the patients

	Polymyositis		Dermatomyositis		
	
	Pt1	Pt2	Pt1	Pt2	Pt3
CX3CL1					
Infiltrated mononuclear cells	++	+++	+++	++++	+++
Endothelial cells	++	++	+++	+++	+++
CX3CR1					
CD4^+ ^T cells	+	+	+	+	+
CD8^+ ^T cells	++++	+++	++++	+++	++++
CD68^+ ^macrophages	+++++	+++++	+++++	+++++	+++++

Muscle tissue sections were scored: -, no staining; +, few of the cells positively stained; ++, some (less than one-half) of the cells stained; +++, approximately one-half of the cells stained; ++++, more than one-half of the cells stained; +++++, most cells stained.

### Expressions of CX3CL1 and CX3CR1 in lung with ILD in PM and DM patients

**Figure 3:**
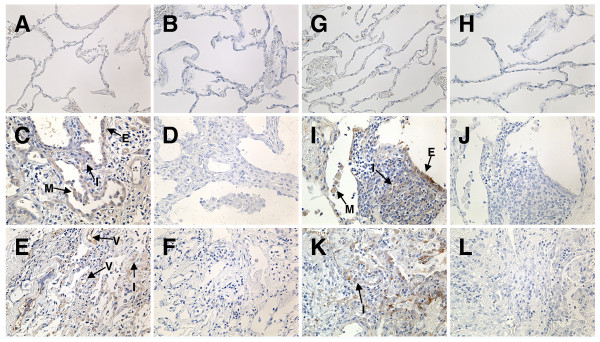
**CX3CL1 and CX3CR1 expressions in lung with ILD in polymyositis patients**. Lung specimens from **(A, B, G, H) **controls and **(C to F and I to L) **polymyositis (PM) patients with ILD were stained with anti-CX3CL1 mAb (A, C, E), anti-CX3CR1 antibody (G, I, K), or isotype-matched control antibodies (B, D, F, H, J, L). Arrows indicate the positive infiltrated mononuclear cells (I), alveolar macrophages (M), vascular endothelial cells (V), and epithelial cells (E). Original magnification, ×400.

CX3CL1 and CX3CR1 expressions in the lung tissue with ILD of PM patients and of DM patients were also analyzed by immunohistochemistry. CX3CL1 was expressed on the infiltrated mononuclear cells, alveolar macrophages, epithelial cells, and endothelial cells in those with PM (Figure 3C and 3E). CX3CR1 was expressed on the infiltrated cells, alveolar macrophages, and epithelial cells in those with PM (Figure 3I and 3K). CX3CL1 and CX3CR1 were also similarly expressed in the lung with ILD of DM patients (data not shown). In contrast, lung tissues of control samples expressed virtually no CX3CL1 and CX3CR1 (Figure 3A and 3G, respectively). Table 4 shows the expression of CX3CL1 and CX3CR1 in the lung tissue from each of the patients.

**Table 4:** Expression of CX3CL1 and CX3CR1 in the lung tissue from each of the patients

	Polymyositis			Dermatomyositis		
	
	Pt1	Pt2	Pt3	Pt1	Pt2	Pt3
CX3CL1						
Infiltrated mononuclear cells	50.8	20.6	12.1	42.3	19.8	13.9
Alveolar macrophages	79.6	71.1	58.3	78.1	53.4	78.6
Epithelial cells	59.5	34.6	53.9	48.3	31.3	45.3
Endothelial cells	22.1	23.6	17.0	17.9	13.8	9.4
CX3CR1						
Infiltrated mononuclear cells	31.8	20.8	23.4	33.0	21.1	44.2
Alveolar macrophages	77.0	70.4	47.0	73.0	54.1	68.2
Epithelial cells	49.5	72.6	59.8	76.5	39.6	38.9

Percentage frequency of CX3CL1 and CX3CR1 expression in each cell type in each of the patients.

### Serum levels of soluble CX3CL1 in patients with PM and with DM

**Figure 4:**
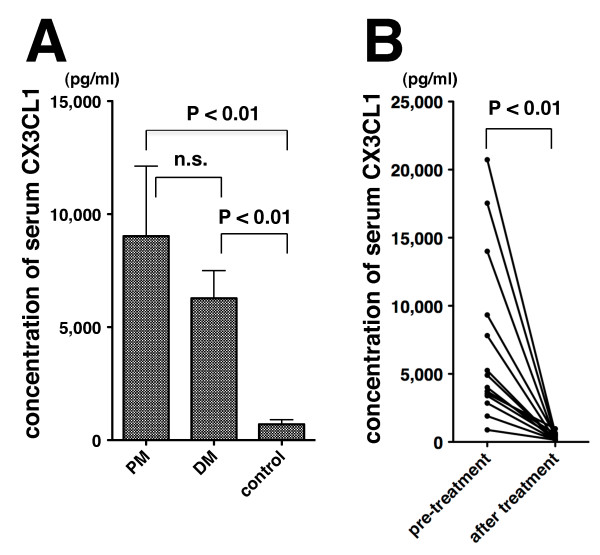
**Serum level of CX3CL1 in patients with polymyositis, patients with dermatomyositis and healthy controls**. Serum samples were collected from polymyositis (PM) patients (*n *= 7), dermatomyositis (DM) patients (*n *= 22) and healthy controls (*n *= 20). **(A) **Concentration of serum soluble CX3CL1 measured by ELISA. Values are mean ± standard error of the mean. **(B) **CX3CL1 levels before and after treatment in PM and DM patients (*n *= 14; three PM patients and 11 DM patients). Symbols joined by a solid line represent data from an individual subject.

We examined the concentrations of serum CX3CL1 in patients with PM and in DM patients, as well as in healthy controls. Serum CX3CL1 in PM patients and DM patients, who had an active newly diagnosed form or flaring up of the disease, was markedly increased compared with that in healthy controls (*P *< 0.01) (Figure 4A). Although the serum CX3CL1 in PM patients tended to be higher than that in DM patients, the difference was not statistically significant (Figure 4A).

To evaluate the effect of treatment on serum CX3CL1, the serum CX3CL1 levels of 14 traceable patients with PM and with DM were analyzed before and after treatment. All patients were treated with oral corticosteroids and/or immunosuppressants, and were in remission after the treatment. In all of the patients, the CX3CL1 level was decreased after treatment (*P *< 0.01) (Figure 4B). Although serum CX3CL1 was also compared between the patients with ILD and without ILD, CX3CL1 levels were not significantly different (serum concentration of CX3CL1: patients with ILD, 7,457.9 ± 1,341.9 pg/ml; patients without ILD, 5,960.9 ± 2,376.3 pg/ml).

**Figure 5:**
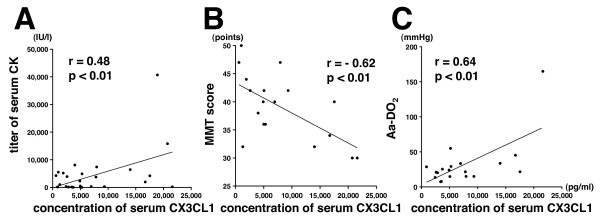
**Correlation of serum CX3CL1 level and the disease activity of polymyositis and dermatomyositis**. Correlations of the level of CX3CL1 with **(A) **serum creatinine kinase (CK) level (*n *= 29), **(B) **manual muscle testing (MMT) score (*n *= 18) in patients with polymyositis (PM) and patients with dermatomyositis (DM), and **(C) **the alveolar-arterial oxygen pressure difference (AaDO_2_) (*n *= 19) in patients with PM and with DM complicated by ILD. Each point represents an individual sample.

To determine whether the serum CX3CL1 level could be a biomarker of the disease activities of PM and of DM, we examined the correlation between serum CX3CL1 and serum CK or MMT score, which are thought to be markers of myositis activity. As shown in Figure 5A and 5B, serum CX3CL1 in patients with PM and patients with DM was significantly correlated with CK (*r *= 0.48, *P *< 0.01) and with MMT score (*r *= 0.62, *P *< 0.01). Among the 29 patients, 19 patients exhibited complication of ILD. The correlation between the serum CX3CL1 level and AaDO_2 _calculated from arterial blood gas [(713 × FiO_2_) - (PaCO_2_/0.8) - PaO_2_] was also analyzed in the patients with ILD. Serum CX3CL1 was significantly correlated with AaDO_2 _in patients with PM and DM with ILD (*r *= 0.64, *P *< 0.01) (Figure 5C).

## Discussion

In this study, we found that CX3CL1 was expressed on infiltrated mononuclear cells and endothelial cells, and that its corresponding receptor, CX3CR1, was expressed on infiltrated inflammatory cells in muscle and lung with ILD in PM patients and DM patients. The serum CX3CL1 level was markedly increased in patients with PM and patients with DM, and was correlated with the titer of serum CK and the MMT score, and also with the AaDO_2 _in patients with ILD complication. These results suggest that interaction between CX3CL1 and CX3CR1 might play an important role in inflammatory cell migration into muscle, as well as lung with ILD, in patients with PM and patients with DM; furthermore, the level of serum CX3CL1 could be a biomarker of disease activity.

Chemokines are thought to play an essential role in inflammatory cell migration into inflamed tissues. In this study, we showed that CX3CL1 was expressed on infiltrated mononuclear cells and endothelial cells in the muscle of PM patients and DM patients. Cytotoxic T cells, including CD4^+ ^and CD8^+ ^T cells, have been reported to invade the muscle fibers in PM patients and DM patients [1], while we previously reported that peripheral blood CX3CR1^+ ^T cells express type 1 cytokines and cytotoxic molecules [12,13]. In addition, CX3CR1 is expressed on peripheral blood monocytes [14]. Indeed, CD4^+ ^and CD8^+ ^T cells and macrophages in the inflamed muscle of PM patients and DM patients expressed CX3CR1. We therefore consider that the interaction between CX3CL1 and CX3CR1 might induce migration of cytotoxic T cells and macrophages, which is partly involved in the pathogenesis of the diseases, into affected muscle. Moreover, it was reported that CX3CL1 provides survival signals and costimulates the production of proinflammatory cytokines and the release of granules [20]. CX3CL1 therefore contributes not only to inflammatory cell accumulation but also to stimulation in the muscle. Our earlier data showed that CX3CL1 and CX3CR1 were expressed in murine EAM and that inhibition of CX3CL1 ameliorated myositis [16]. Taken together, these findings suggest that CX3CL1 might play an important role in myositis, and therefore that blockade of CX3CL1 might be therapeutically beneficial for patients with PM and patients with DM.

The level of serum soluble CX3CL1 obtained from patients with active PM and DM was increased, and the CX3CL1 level was markedly decreased after immunosuppressive therapy. Moreover, the CX3CL1 level was significantly associated with the serum CK level and MMT score, which are indicative of myositis [21,22]. The serum CX3CL1 level could therefore be a biomarker of disease activity. Increased CX3CL1 in inflamed muscle might induce the migration of CX3CR1-expressing inflammatory cells - including type 1 cytokines, and cytotoxic molecule-expressing T cells and macrophages - into the tissue, and these cells, in turn, express TNFα and IFNγ, which induce additional CX3CL1 expression on endothelial cells [23] and also on recruited inflammatory cells. As a result of these expansive cascades, CX3CL1 is highly expressed in the inflamed muscle, and the serum CX3CL1 level might consequently be increased.

We also found that the serum CX3CL1 level in both PM patients and DM patients with the complication of ILD was significantly associated with the AaDO_2_. CX3CL1 was expressed on the infiltrated mononuclear cells, endothelial cells, alveolar macrophages, and epithelial cells in the affected lung tissues. CX3CR1 was also expressed on infiltrated mononuclear cells, alveolar macrophages, and epithelial cells. The interaction between CX3CL1 and CX3CR1 might be involved in the inflammatory cell infiltration into lung tissue. In addition, CX3CL1 may stimulate CX3CR1-positive cells for survival, production of cytokines, and release of granules in the lung. Serum CX3CL1 was not significantly different between the patients with ILD and those without ILD. Not only ILD, but also muscle inflammation, may affect the serum CX3CL1 level.

Serum CCL17 has been reported to be increased in DM patients with ILD [24]. Serum levels of CCL2, CCL3, CCL8, CXCL10, CXCL11, IL-6, and IL-18 have also been shown to be correlated with the disease activity of DM [25-27]. In this study, we showed that serum CX3CL1 correlated with the serum CK and MMT score in patients with PM and patients with DM, and also with the AaDO_2 _in the patients complicated by ILD. Serum CX3CL1 was also increased in asthma, scleroderma, and rheumatoid arthritis [28-30]. Consequently, although CX3CL1 is not a disease-specific marker, upon diagnosis of PM or DM the serum level of CX3CL1 could be a useful marker of disease activity of myositis, as well as for the complicated ILD.

There are some limitations with CK and MMT as markers of disease activity, as they may not always follow disease activity. More detailed validation - such as disease activity tools for use in myositis clinical trials, as described by the International Myositis Assessment and Clinical Studies Group [31-33] - may be needed in future study. Also, samples in this study were retrospectively analyzed; selection biases may therefore influence the results.

## Conclusion

The interaction between CX3CL1 and CX3CR1 might contribute to the pathogenesis of PM and of DM, and these could be appropriate molecules for therapeutic targeting. The serum level of CX3CL1 could be a surrogate marker of disease activity.

## Abbreviations

AaDO_2_: alveolar-arterial oxygen pressure difference; CK: creatinine kinase; DM: dermatomyositis; EAM: experimental autoimmune myositis; ELISA: enzyme-linked immunosorbent assay; IFN: interferon; IL: interleukin; ILD: interstitial lung disease; mAb: monoclonal antibody; MMT: manual muscle testing; PBS: phosphate-buffered saline; PM: polymyositis; TNF: tumor necrosis factor.

## Competing interests

The authors declare that they have no competing interests.

## Authors' contributions

FS participated in the design of the study, carried out the experiments and statistical analysis, and drafted the manuscript. TK, YM, KI, ME, SH, and TO assisted carrying out the experiments and manuscript preparation. HM and TI assisted in data interpretation and manuscript preparation. NM and TN conceived of the study, participated in its design and coordination, and helped to draft the manuscript. All authors read and approved the final manuscript.
